# Collagenase Promotes the Cellular Responses to Injury and Wound Healing In Vivo

**Published:** 2005-05-17

**Authors:** Kathleen N. Riley, Ira M. Herman

**Affiliations:** Department of Physiology, Tufts University School of Medicine, Boston, Mass

## Abstract

**Objective:** This study focuses on the growth-promoting and migration-enhancing role that Clostridial collagenase plays in vitro and in vivo. **Methods:** For in vitro studies, biosynthesized extracellular matrices were treated with purified Clostridial collagenase, nonspecific proteases, or buffer controls. Keratinocytes were subsequently plated upon these matrices in the presence or absence of Clostridial collagenase and/or heparin-binding epidermal-like growth factor, and cell proliferation and migration were quantified. To examine the effects of Clostridial collagenase in vivo, we performed a double-blind study of full-thickness wounds on the backs of Yucatan Micropigs, testing the effects of purified Clostridial collagenase, Regranex (PDGF-BB), and Solosite (carboxymethyl cellulose) on wound healing. **Results:** *In vitro studies:* Matrix pretreatment with Clostridial collagenase stimulates a 2-fold increase in proliferation and postinjury migration; when Clostridial collagenase and/or heparin-binding epidermal-like growth factor are added to the growth media, there is an additional doubling of growth and migration, yielding approximately 5-fold enhancement of keratinocyte proliferation and migration. Papain-urea treatment under similar conditions results in a 50% decrease in cell number over a 1-week time course. *In vivo studies:* By all parameters measured, including granulation tissue formation, inflammation, re-epithelization, and time to wound closure, purified Clostridial collagenase was superior (analysis of variance, *P* > .05) to other treatments tested. **Conclusion:** On the basis of these findings, we concluded that Clostridial collagenase stimulates keratinocyte cellular responses to injury in vitro and may represent a novel therapeutic approach for promotion of wound healing in vivo.

The keratinocyte basement membrane serves as a scaffold and a macromolecular signaling matrix responsible for regulating cell behavior during development, adult life, and wound healing. Synthesized and organized by keratinocytes, matrix components play a pivotal role in orchestrating epithelial proliferation,^1^ adhesion,^2^ and migration,^3^ including the cellular responses to injury. Glycoproteins and proteoglycans not only provide a physical scaffold for epithelial cells but also can bind and sequester signaling molecules, including members of the epidermal growth factor (EGF),^4^ fibroblast and keratinocyte growth factor,^5^,^6^ and transforming growth factor families.^7^ Keratinocytes transduce signals from these matrix-bound or soluble growth factors through their high-affinity tyrosine and serine/threonine kinase growth factor receptors, thereby completing this biochemical signaling cascade.

Keratinocyte responsiveness to the extracellular matrix involves controlling the expression, activation, and localization of the receptors that transduce matrix-associated signals. For example, the activation and relocation of integrin receptors in response to injury is crucial to the initiation of wound healing. In the wound area, keratinocytes come in contact with the newly exposed dermal matrix, which contains Type I collagen and laminin 5 not found in the intact keratinocyte basement membrane, typically composed of collagen IV, laminin 1, fibronectin, and other molecules.^8^,^9^ The newly exposed dermal matrix components bind and activate the α2β1 integrin receptor, causing its relocation to the site of injury, thus allowing keratinocytes to adhere to the exposed dermal matrix and begin migration into the wound area.^10^ Expression of αVβ5 integrin is also induced at the wound edge, and likewise promotes migration.^11^ Interestingly, knockout mice lacking the β1 integrin subunit show abnormal keratinocyte morphology and are highly deficient in wound healing because of defects in adhesion, migration, and proliferation.^12^

In addition to the modifications seen in the integrin receptor repertoire during wound healing, injured keratinocytes release growth factors and matrix metalloproteinases (MMPs) to stimulate their integrin-mediated migration.^13^,^14^ Growth factors, such as heparin-binding epidermal growth factor (HbEGF), are produced by keratinocytes in a membrane-tethered form. In response to injury, HbEGF is cleaved and released by extracellular MMP-3, or Stromelysin I.^15^ Keratinocytes also produce MMP-1, MMP-2 and MMP-9, all of which help to remodel the matrix, allowing cells to migrate into and close the wound.^9^,^16^–^18^ MMP production is stimulated by the presence of target substrates and growth factors,.^9^,^16^,^19^–^21^ and MMP-1 can also be bound by integrin α2β1 for targeted collagen degradation.^22^ When healing is completed and the wound is closed, the remodeled matrix signals through integrins to downregulate keratinocyte MMP and growth factor production.^9^

Under normal circumstances, the dynamic exchange between keratinocytes and matrix results in complete wound closure and regeneration of the dermis and epidermis. However, some wounds fail to heal normally and become chronic. Chronic wounds exhibit a healing profile different from that for normal acute wounds,^23^,^24^ remaining in an inflamed state for protracted periods of time. Chronic wounds are frequently exacerbated by intrinsic factors such as excess inflammatory cytokines and poor vascularization/ischemia.^25^ The deregulation of integrins^26^ and overabundance of MMPs,^27^–^29^ as well as the lack of tissue-specific inhibitors of MMPs,^30^ can also give rise to chronic wounds. These wounds almost always require clinical intervention in order for healing to occur.

Because of the fluctuating balance of activating and inhibiting signals that are induced during wound healing, we question whether exogenous application of factors known to regulate normal healing might accelerate the acute healing process, or activate the healing of chronic wounds. We have previously shown that collagenase from *Clostridium* bacteria promotes the migration and proliferation of vascular endothelial cells and keratinocytes^31^ after injury, with an efficiency more than twice that of mammalian collagenase. While mammalian enzymes cleave collagen at one site to produce one-quarter and three-quarter size fragments,^32^ Clostridial collagenase cleaves all 3 helical domains to produce several breakdown products.^33^,^34^

There has also been a great deal of interest in the papaya-derived protease papain, initially reported as a wound treatment 4 decades ago.^35^ Research is still ongoing into the precise role this enzyme may have in promoting healing,^36^ though it is currently classified as an enzymatic debriding agent. We have examined its effects on keratinocytes in vitro to determine whether its clinical efficacy may be due to the promotion of keratinocyte proliferation and/or motility. We have also revisited the Clostridial collagenase experiments on keratinocytes, and expanded our investigation to include an in vivo wound healing assay on miniature swine. In our in vivo study, we have compared Clostridial collagenase with 2 commercially available preparations, Regranex, the active ingredient of which is PDGF-BB, and Solosite, a cross-linked carboxymethyl cellulose–based hydrogel that maintains a moist healing environment, as well as a sterile gauze negative control. Overall, we hope to clarify the role that Clostridial collagenase may play as a wound healing agent.

## METHODS

### Cells

Epithelial keratinocytes isolated from normal human epidermal keratinocyte (NHEK) were purchased from Clonetics (Walkersville, Md) and cultured in complete Keratinocyte Growth Medium (KGM, Clonetics) in 175 cm^2^ tissue culture flasks (Costar) as previously described.^31^ Cells were passaged with trypsin, quenched with medium containing 5% bovine calf serum, rinsed in phosphate-buffered saline, and replated in KGM at a ratio of 1:4 for cell propagation and at specified cell numbers for all experiments. Cells were fed every other day by complete media exchange with fresh KGM. In all experiments where soluble factors were added to the growth media, these factors were added at the time of cell plating, and replenished as cells were fed on alternate days. HbEGF was obtained from Oncogene, San Diego, Calif.

### Matrix Preparations

Matrix preparations were made as previously described.^31^ Briefly, capillary endothelial cells at 10 days postconfluence were released from their matrix with DOC buffer (0.5% sodium deoxycholate, 0.02 M Tris-Cl [pH 8.0], 0.015 M NaCl, 0.001 M EGTA [pH 7.0], 0.001 M phenylmethyl sulfonyl fluoride), and the remaining matrix was washed with phosphate-buffered saline. Enzymes used for matrix digestions were diluted in calcium-buffered saline, for a total of 250 μL per well in a 24-well plate. Digestions were performed at 37°C for 1 hour in a cell culture incubator. Following digestion or control treatment with calcium-buffered saline alone, the matrix was first washed with phosphate-buffered saline and then allowed to equilibrate in KGM for 15 minutes at 37°C. The KGM was removed, and NHEK cells were released from flasks with trypsin, counted, and plated onto the matrix in fresh KGM. Earlier published experiments indicate that the enzyme doses used for matrix treatment do not affect the plating efficiency of the keratinocytes.^31^

### Enzymes

Enzymes used for extracellular matrix digestion were purified collagenase from *Clostridium*, crude collagenase, and clostripain (Advanced Biofactures Corp, Lynbrook, NY), as in previous experiments.^31^ A mixture of papain and 1% urea (Beckton Dickinson, Sparks, Md) was also tested,^36^–^39^ with or without the addition of chlorophyllin (Beckton Dickinson).^40^–^42^ All treatment doses are given in standard units of enzymatic activity.

### Cell proliferation assay

Growth assays were performed as previously described.^31^ Briefly, NHEK cells were released from the matrix with trypsin, diluted in Isoton II electrolyte buffer (Beckman, Fullerton, CA) for counting in a Coulter Counter model ZF (Coulter Electronics, Miami, Fla). Experimental conditions were plated in duplicate, and each sample was counted twice. Each condition was tested in at least 3 separate experiments. Data were recorded manually and analyzed in Microsoft Excel (Microsoft, San Jose, Calif).

### Cell migration assay

For motility studies, matrix was prepared on 10 mm^2^ glass cover slips (Corning, Big Flats, NY) as described above. NHEK cells were plated at confluence on the resultant matrix and allowed to attach overnight. Cell monolayers were injured with a fire-polished pasteur pipette to create a narrow scratch wound. The wounded populations were monitored through time-lapse imaging, as previously described.^43^ Relative motility was calculated by comparing the change in area covered by cells in the same sized viewing field over the same period of time for different treatment conditions.

### Wound healing study

A full-thickness wound healing study on swine was conducted by North American Science Associates (NAMSA), Northwood, Ohio, in accordance with the National Institutes of Health guidelines for the treatment of laboratory animals. Treatments tested were (1) *Clostridium* bacterial collagenase in ointment, (2) Regranex gel, (3) Solosite gel, and (4) dry sterile gauze as a negative control. The study was conducted on 8 healthy female adult Micro-Yucatan Miniature swine (*Sus scrofa domesticus*), selected on the basis of their use in other published research and their similarity of wound healing to humans. Each animal received eight 2-cm diameter full-thickness wounds on its back while under general anesthesia. Pressure was applied to stop bleeding before any treatment or dressing was affixed on Day 0. Two wounds on each pig were dressed with each of the 4 treatment conditions, with locations (cranial to caudal) randomized. Clostridial collagenase was prepared at 1000 U/g of ointment. Standard commercial preparations of Regranex and Solosite were used. Ointments were applied at 0.25 g/d, according to the directions for use of the commercial products. Treated wounds were covered with OpSite Flexgrid, a transparent wound dressing. The dry control wounds were covered with sterile gauze. All bandages and dressings were changed daily. The gauze dressings were removed carefully by a veterinarian, who noted minimal disruption to the wounds due to moisture from wound exudation. Observations were recorded daily for each wound, such as the diameter, color, exudation, and epithelization, in a double-blinded manner. Wound area was calculated from tracings made on transparent plastic during daily examinations. Following daily observations, the wound area was gently rinsed with sterile saline and dried with sterile gauze to permit adhesion of the new dressing. Vigorous flushing of the wound itself was not performed.

At the conclusion of the study, each wound was excised, with a 1 to 2 cm border of intact skin, for histological processing. Each sample was fixed in 10% neutral buffered formalin, embedded, sectioned, and stained with hematoxylin and eosin. Two sections were taken from each wound sample: one across the central diameter of the wound and one parallel section from the side halfway to the edge of the original defect. Epithelization was recorded from the central section of each wound as the percentage of the defect covered by well-defined dermal epithelium.

## RESULTS

### In vitro proliferation

To simulate the in vivo basement membrane of keratinocytes, we used the biomatrix generated by confluent capillary endothelial cells, which has been shown to contain fibronectin, laminin, and collagen of types I, III, and IV.^44^ Treatment of the endothelial-produced biomatrix with purified bacterial collagenase prior to plating caused an approximate doubling in keratinocyte proliferation as compared to cells on untreated matrix (Table 1). The addition of HbEGF^14^,^31^,^45^ to the culture media was also tested in proliferation assays. The addition of 0.1 ng/mL HbEGF to cells on untreated matrix caused a 1.4-fold increase in proliferation over controls after 7 days, but when added to cells growing on collagenase-digested matrix, proliferation nearly tripled. Treatment of the matrix with crude collagenase, which contains other nonspecific proteases, caused only a slight increase in cell number over controls. Clostripain, a purified nonspecific protease, produced an even smaller increase in proliferation. Pretreatment of the matrix with urea and papain,^36^,^38^,^46^ with or without chlorophyllin, diminished cell proliferation to half of control numbers. We next asked whether addition of collagenase to the growth media would affect proliferation, alone or in combination with matrix pretreatment (Table 1). On untreated matrix, incubating cells with 16 U/mL soluble collagenase in the growth medium resulted in a 1.6-fold increase in proliferation. When cells were grown on matrix pretreated with collagenase, and incubated with the same dose of soluble enzyme, total proliferation was 4.5 times that for control, and nearly double the result seen with treated matrix alone.

### In vitro motility

**Figure 1:**
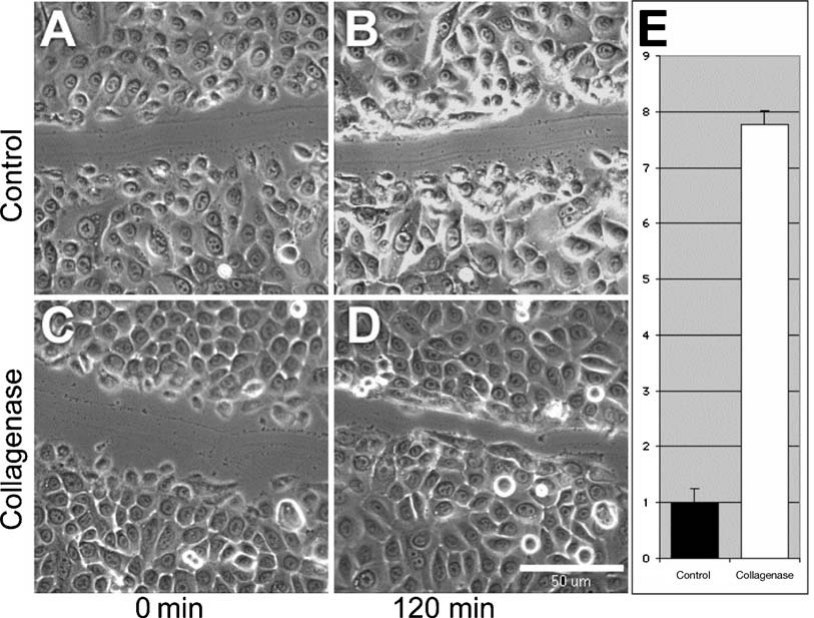
Keratinocyte motility in vitro. This figure shows the relative ability of keratinocytes to spread and crawl to close a narrow in vitro wound. A population grown on untreated matrix in normal media (A and B) and a population of the same density on matrix treated with collagenase and with collagenase present in the media (C and D) are shown just after wounding (A and C) and 120 minutes incubation postwounding (B and D). Scale bar equals 50 μm. Time lapse digital images, available as Quicktime movies, can be viewed at http://www.journalofburnsandwounds.com. Panel E shows the quantified relative motility of control (*n* = 2) and treated (*n* = 2) populations during the initial 30 minutes after wounding, with the control set equal to 1. Error bars represent the standard error of the mean (*P* < .01, Student *t* test).

To assess the effect of bacterial collagenase on cell motility, we used digital imaging microscopy to monitor the rate at which a confluent cell monolayer is able to close a narrow wound. In all conditions, keratinocytes at the wound edge first elaborate a membrane fan, then detach the trailing edge, and finally pull the nucleus and cell body forward toward the leading edge. As this happens, cells further from the wound edge spread to fill the newly available space. On matrix pretreated with collagenase, the cells at the leading edge extend a much longer membrane fan before translocating the rest of the cell, thus covering a greater distance in a shorter period of time (Figs 1C and 1D). Overall, keratinocyte motility was increased nearly 8-fold over controls when assayed on treated matrix, in the presence of HbEGF and soluble collagenase (Fig 1E). Time-lapse digital images can be viewed as Quicktime movies at http://www.journalofburnsandwounds.com.

### In vivo

**Figure 2:**
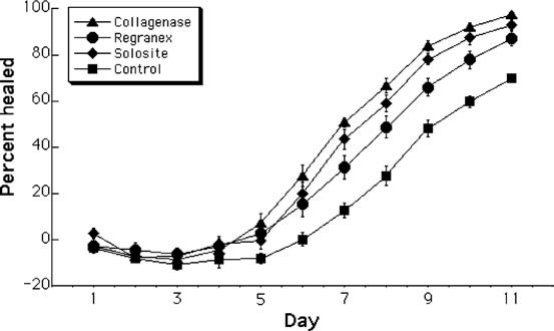
Percentage healing of in vivo wounds. This chart shows the average percentage of wound closure, based on the original wound area, on each day of the experimental treatment with Clostridial collagenase, Regranex, Solosite, or a dry, sterile gauze control (*n* = 16 for each condition). Error bars represent the standard error of the mean. Beginning on Day 6, when healing began to result in a decreased wound size, there was a statistically significant difference among the treatment groups (analysis of variance, *P* < .05), and between each individual treatment and the sterile gauze control (Tukey test, *P* < .05). From Day 7 onwards, the difference between collagenase and each of the other 2 treatments is also significant (Tukey test, *P* < .05), though Solosite and Regranex did not differ significantly from each other.

**Figure 3:**
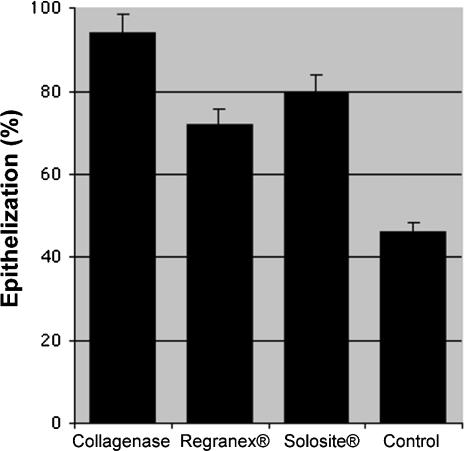
Re-epithelization of in vivo wounds. This chart shows the percentage of re-epithelization of in vivo wounds treated with collagenase, Regranex, Solosite, and a dry, sterile gauze control (*n* = 16 for each condition), based on histological samples prepared at the conclusion of the study on Day 11 (*P* < .01, analysis of variance).

**Figure 4:**
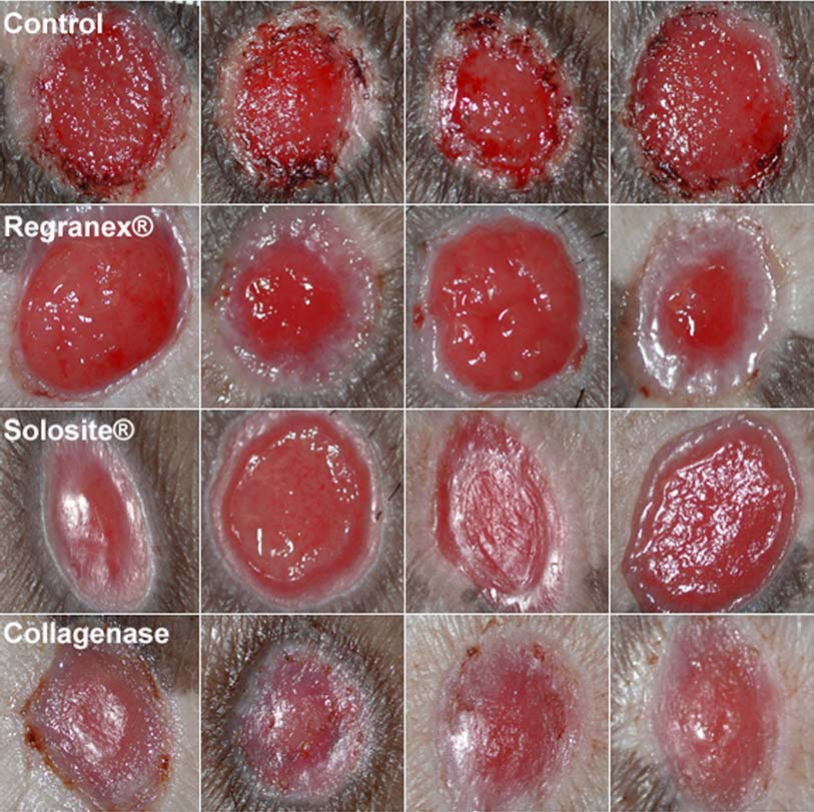
Comparison of in vivo wounds on Day 10. This figure shows 4 wounds from each of the treatment groups, photographed on Day 10 of the study. The wounds treated with Clostridial collagenase show the least fluid exudation and the greatest re-epithelization.

In order to determine whether the enhancement of proliferation and migration seen in vitro would translate into the promotion of healing in vivo, a double-blinded study of full-thickness wounds on Yucatan Micropigs was conducted by NAMSA. Superficial observations of wound color and exudation did not vary markedly among the treatment conditions. Granulation tissue developed more slowly in control wounds than in treated wounds, but no obvious difference was noted between treatment conditions. By analysis of the wound area as a percentage of the original wound, it was found that collagenase accelerates overall healing (Fig 2). The differences among the treatment conditions became statistically significant (analysis of variance, *P* < .05) beginning on Day 6 of the trial, when the wound area began to decrease. Differences between collagenase and Solosite or collagenase and Regranex became significant (Tukey test, *P* < .05) from Day 7 onward. The difference between the collagenase condition and the control condition was significant (Tukey test, *P* < .05) from Day 4. The area of the wound covered by new epithelial tissue in the terminal histology samples was also greater with collagenase treatment than with Regranex, Solosite, or the dry control (Fig 3). This improved re-epithelization was accompanied by a decrease in observed granulation tissue in the collagenase-treated wounds (not shown). The improved condition of the collagenase-treated wounds can be seen in photographs taken on Day 10 after wounding (Fig 4).

## DISCUSSION

Through our experiments, we have reaffirmed that pretreatment of a biosynthesized extracellular matrix by Clostridial collagenase promotes human keratinocyte responses to injury. Collagenase acts through multiple signaling pathways, increasing proliferation and migration in vitro. It is effective via pretreatment of the matrix and/or its presence in the growth media. Interestingly, the effects of collagenase are strongly potentiated by inclusion of HbEGF, which is prevalent in in vivo wounds.^13^,^14^ Through a combination of matrix pretreatment and addition of soluble collagenase and/or HbEGF in the growth media, keratinocyte proliferation can be potentiated 5-fold. Keratinocyte migration following injury in vitro is similarly enhanced. Papain-urea, alone or in combination with chlorophyllin, currently in clinical use for enzymatic debridement of wounds,^36^ inhibited keratinocyte proliferation in vitro, over a wide range of tested doses. Importantly, purified Clostridial collagenase also promoted the cellular responses to injury in vivo, resulting in reduced granulation tissue, increased rates of re-epithelization, and a shorter interval to wound closure.

Pretreatment of biosynthesized extracellular matrix,^47^ which is analogous to the human keratinocyte basement membrane, with Clostridial collagenase promoted cell proliferation (Table 1) and migration (Fig 1). These results are supported by earlier work from this laboratory on vascular endothelial cells migrating in response to injury.^44^,^48^ In addition, endogenous collagenase has been shown to promote keratinocyte migration in vitro.^16^,^49^ Collectively, these results point to the important role that collagenase and other MMPs play in modulating cellular responses to injury and wound healing.

Inclusion of Clostridial collagenase in the growth media, alone or in addition to matrix pretreatment, was also able to stimulate the keratinocyte response to injury (Table 1). Furthermore, HbEGF stimulated keratinocyte proliferation in vitro, as well as potentiated the migration-enhancing effects of Clostridial collagenase (Table 1). It has been shown in vitro that stromelysin, or MMP-3,^15^ is able to cleave HbEGF at the juxtamembrane site, releasing the mature, soluble form of the growth factor to signal in a paracrine or juxtacrine fashion. Interestingly, MMP-3 cleaves HbEGF successfully, but MMP-2 and MMP-9 do not. Other in vitro studies have shown that membrane-type MMP-1 can cleave and activate pro-αV, -3, and -5 integrins.^49^,^50^ Through its processing of the αV integrin subunit, MT-MMP-1 prevents the suppression of the collagen-binding α2β1 receptor by αVβ3,^51^ with the overall effect promoting migration on Type I collagen. These data indicate that membrane-associated growth factors and receptors can be released and/or activated through MMP action. Similarly, by releasing and activating endogenous promoters of growth and migration, Clostridial collagenase may be able to induce the keratinocyte responses to injury.

Our in vivo wound healing study on swine extends our in vitro findings that Clostridial collagenase promotes epithelial cell proliferation and migration by revealing that direct application of purified Clostridial collagenase to full-thickness wounds promotes healing and improves wound health as compared to other commercial preparations tested or untreated controls. There are numerous reports indicating that several types of collagenase have already been tested and are in clinical use as enzymatic debriding agents.^52^–^56^ From the studies using preparations of Clostridial collagenase,^57^,^58^ there is ample evidence to indicate that collagenase not only successfully debrides wounds but also hastens wound closure.^53^ Altogether, these results point to a promising role for collagenase in promoting acute and chronic wound healing.

## Figures and Tables

**Video 1:**
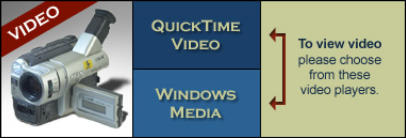
Keratinocytes on untreated matrix crawling in response to injury.

**Video 2:**
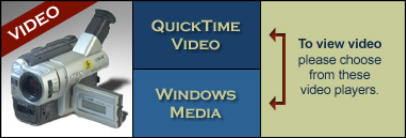
Keratinocytes on collagenase-treated matrix, with collagenase and heparin-binding epidermal growth factor in the media, crawling in response to injury.

**Table 1:** Cell proliferation in vitro, Day 7*

	**Without soluble enzyme**	**With soluble enzyme**
Untreated matrix	1.0	1.6
Purified collagenase	2.3	4.5
Purified collagenase + HbEGF	2.9	4.9
HbEGF alone	1.4	2.6
Crude collagenase	1.2	2.3
Clostripain	1.1	2.0
Papain-Urea/chlorophyllin	0.5	

*HbEGF indicates heparin-binding epidermal growth factor. Results shown are the comparative effects of matrix pretreatment and soluble factors on proliferation. Soluble enzymes are the same as those used for matrix pretreatment; cells plated on untreated matrix received soluble Clostridial collagenase. All soluble enzymes were added at 16 U/mL in the culture media. HbEGF was applied both in the presence and absence of soluble enzyme in the indicated samples. The number of cells in the control condition was set equal to one, and all other results are shown in relation to this control. For each condition, the average of at least 3 separate experiments is shown. All standard deviations are less than 0.05.
